# Study of demographic, clinical, laboratory and electromyographic symptoms in Myasthenia Gravis patients referred to the neurology clinic of Rasoul Akram hospital in 2015

**Published:** 2015

**Authors:** Y Sadri, B Haghi-Ashtiani, B Zamani, FH Akhundi

**Affiliations:** * Rasoul Akram Hospital, Iran University of Medical Science, Tehran, Iran

**Keywords:** demographic characteristics, clinical symptoms, electromyographic, Myasthenia Gravis

## Abstract

**Introduction.** Myasthenia Gravis is an autoimmune disorder, which is clinically a neuromuscular illness that shows itself as muscular weakness and fatigue. The diagnosis of Myasthenia Gravis depends on clinical evaluation, electrophysiological assessment, and autoantibody detection in serum. Known antibodies could be found in about 90% of the patients, which had a causative relation with disease symptoms. Therefore, the purpose of this paper was a survey on demographic features, clinical, laboratorial, and electromyographic signs of patients with Myasthenia Gravis referred to the neurology clinic of Rasoul Akram hospital.

**Materials and methods.** This study was a descriptive cross-sectional one that used an easy sampling method: 54 patients with Myasthenia Gravis who were referred to the neurology clinic of Rasoul Akram were elected in 2015. The patients’ information was recorded in the checklists based on the variables and the data were analyzed by using SPSS software version 21.

**The results.** The demographic and the clinical symptoms data of 54 known Myasthenia Gravis patients, whose diagnosis was made according to the clinical symptoms, electrophysiological findings and autoantibody detection, were analyzed in this paper. There were 31 females (57.4%) and 23 males (42.6%) with an average age of 47.3 years. The average age of diagnosis of Myasthenia Gravis in these patients was 42.8 years. Among the patients, 19 (35.2%) had a hospitalization history because of their disease. Due to laboratory findings, 10 patients (18.5%) had Musk antibody, 34 patients (62.9%) had acetylcholine receptor antibodies and 10 patients (18.5%) had none of these two antibodies. Moreover, in electromyographic findings, 38 patients (70.37%) had positive findings and 16 patients (29.6%) had normal findings.

**Discussion and Conclusion.** Due to the chronic nature of this disease, and its rising trend, educating the people for the early detection of the disease, was necessary as soon as possible so as they would be treated and an acceptable life would be provided for them.

## Introduction

Myasthenia Gravis is a type of autoimmune disease of nerve-muscle junction, which, in many cases, due to a kind of antibody against acetylcholine receptor these antibodies were created in the serum [**1**-**4**]. It is found in 80 to 90% of the patients with generalized Myasthenia and it only exists in a small percentage of healthy individuals. A group of patients with Myasthenia Gravis who lack such antibodies is called seronegative [**1**,**3**,**5**]. Several studies showed significant differences between the two groups - seronegative and seropositive [**1**,**6**,**8**]. Some sporadic studies have shown that the severity of Myasthenia Gravis in women is more than in men [**6**], and this difference is more pronounced in the seronegative group. Moreover, it seems that an increase in age and female hormones may predispose a person to a type of Myasthenia Gravis seropositive and not the type of seronegative [**6**]. Other studies have shown that patients with seronegative and seropositive have similar symptoms and they only have differences in disease severity and age of onset [**6**], although some studies have not reported a significant difference in disease severity in patients with seronegative and seropositive myasthenia Gravis [**3**]. 

Myasthenia Gravis is an autoimmune neuromuscular disease, which is diagnosed with weakness and fatigue of skeletal muscle. The main problem of this disease is in the reduction of acetylcholine receptors at the neuromuscular junction. The disease has no definite cure, but treatments are now very effective. The outbreak of the disease is of 2-7 cases per 10,000 people. The highest incidence of the disease in women is in the second and third decade and in men is in the sixth and the fifth decade. Female to male ratio is 3: 2 [**8**]. An accurate estimation of the incidence of Myasthenia Gravis is of about 30/ 1000000. The incidence in children and adolescents aged 0-19 years is between 1.0 and 5.0 in 1 million [**3**].

A wide age distribution of this disease and the disability that limits the patients’ function, during the 4-7 years, represent the specific aspects of this disease. The important point is the lack of a timely diagnosis, and the risks of taking certain medications, and surgical procedures and anesthesia, which in turn, lead to the respiratory crisis and mortality risk, in the case of non-recognition. The clinical course of Myasthenia Gravis treatment is variable, and spontaneous remissions are still rare [**4**].

The first step for diagnosis includes the health status of the individual, physical, and neurological examinations. The doctor should be looking for abnormalities in eye movements or eye muscle weakness. If the doctor suspects Myasthenia Gravis, there are several tests to confirm.

- Certain blood test: finds immune system molecules or acetylcholine receptor antibodies. In most patients, the amount of these antibodies is abnormally high

- Edrophonium test: an amount of chloride Edrophonium is injected intravenously. This drug causes the degradation of acetylcholine, which is blocked, and temporarily increases the amount of acetylcholine at the neuromuscular junction. In patients, chloride Edrophonium recovers the weakness.

Other methods include nerve conduction study. By using this method, when the nerves are stimulated repeatedly, the muscle weakness is characterized.

Electromyography Test (EMG): With this method, the electrical potential of muscle cells is measured and the disorder in nerve transmission to the muscle is diagnosed. The muscle fibers in Myasthenia Gravis do not respond well to repeated electrical stimulation. 

Computed tomography (CT) is used for the detection of abnormal thymus gland or a tumor in the thymus gland. The lung function test measures the breathing power [**7**,**9**].

The treatment: anticholinesterase factors such as neostigmine and pyridostigmine

Immunosuppressive drugs, including cyclosporine, Prednisone, and Azathioprine

Thymectomy: surgery for the removal of the thymus gland

Plasmapheresis: is a procedure in which abnormal antibodies are removed from the blood [**5**,**9**].

Given the importance of the early diagnosis of the disease, through clinical and laboratory symptoms, and the acceleration of their treatment, and reducing the effects of this disease, it was decided to investigate the demographic characteristics, clinical and laboratory symptoms in patients with Myasthenia Gravis who referred to the neurology clinic of Rasoul Akram hospital, thus taking a step towards improving the conditions of these patients.

**Research methodology**

The study was conducted in a cross-sectional method, in which the records of 50 patients with Myasthenia Gravis who referred to the neurology clinic of Rasoul Akram hospital in 2015 were studied. MG diagnosis is characterized by common clinical features of the disease, such as ptosis, double vision and dysphagia and proximal limb weakness, or some respiratory discomfort with some attacks on them. More MG clinical diagnosis results of electrophysiological tests, including repetitive nerve stimulation (RNS), or Electromyography are supported from a single fiber (SFEMG) and the existence of Acetylcholine receptor antibody is used. It should be noted that two neurologists examined all the patients and they agreed with the diagnosis of Myasthenia Gravis. Checklists included gender, age, history of hospitalization, clinical symptoms such as paresthesia, muscle weakness, gait problems, difficulty in breathing, double vision, dysphagia and speech difficulty, ptosis and laboratory variables such as blood tests (acetylcholine receptor antibody, MUSK antibodies) and RNS, SFEMG. Then, the data were entered into SPSS 21 software, and, Chi square test was used to compare the qualitative variables, and the T-test was used to compare quantitative variables.

Using SPSS 21 software, 54 samples were entered and the Chi square test was used to determine the frequency percentage and comparison, the average was calculated to compare the qualitative variables and the quantitative variables, and the T-test was used to compare these variables.

**Findings**

According to the clinical evaluation, 54 patients were entered into the study with the definitive diagnosis of Myasthenia Gravis. The average age of the patients was 47.3 years with a standard deviation of 18.28. The youngest patient was 16 years old and the oldest patient was 85 years old.

The age of diagnosis had the average of 42.8. There were 2 patients (3%) at an age below 20 and both were men. There were 22 patients (40%), of whom 7 cases (12.96%) were males and 15 were females (27%) in the age range of 20-40 years old. There were 18 patients, including 3 males (5.5%) and 15 females (27%) in the range of 40-60 years old, and 12 patients - 10 males (18.5%) and 2 females (3%) in the age range above 60. 

The average age of women was 42.32 ± 14.11 years and the average age for men was 54.13 ± 21.22 years. According to the available data, a significant statistical difference could be found between the average age of men and women (P = 0.026). On the other hand, the average age of diagnosis in women was 38.03 ± 14.76 years and for men 49.26 ± 22.38 years, that, according to the available data, the difference was statistically significant between the average age of diagnosis in men and women (P = 0.044). In fact, men in the older ages will have the disease and in later ages, symptoms emerged and the diagnosis will be done at a later age.

Of the patients, 31 (57.4%) were females and 23 (42.6%) were males. Among the patients, 19 (35.2%) had a history of hospitalization and 35 (64.8%) had no history of hospitalization. 

**Table 1:** Percent of negative and positive cases for different ailments

	negative	positive
paresthesia	50 (92.6%)	4 (7.4%)
weakness	21 (38.9%)	33 (61.1%)
Gait disorder	48 (88.9%)	6 (11.1%)
respiratory	43 (79.6%)	11 (20.4%)
diplopia	25 (46.3%)	29 (53.7%)
dysphagia	40 (74.1%)	14 (25.9%)
ptosis	19 (35.2%)	35 (64.8%)

4 patients (7.4%) had paresthesia and 50 patients (92.6%) did not suffer from paresthesia. 33 patients (61.1%) had muscle weakness and 21 patients (38.9%) had no muscle weakness, of whom 13 cases (24%) had ocular Myasthenia Gravis. Among the cases, 6 people (11.1%) had gait problems, and 48 patients (88.9%) did not mention it. Respiratory crisis was seen in 11 patients (20.4%) and 43 patients (79.6%) did not show such a problem. Among the patients, 29 (53.7%) had diplopia and 25 (46.3%) did not suffer from diplopia. Dysphagia was seen in 14 patients (25.9%) and it was not seen in 40 patients (74.1%). Among the cases, 35 patients (64.8%) had ptosis, and 19 patients (35.2%) did not have ptosis (**Table 1**).

**Fig. 1:**
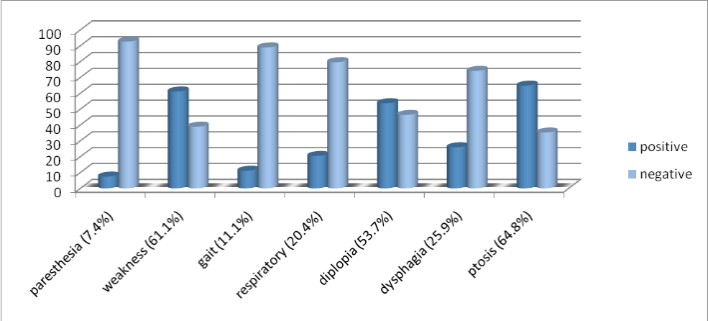
Positive and negative percent of different ailments

Thymectomy was recorded in 22 patients (40.7%) and it was not recorded in 32 patients (59.3%).

According to the laboratory findings, among the cases, 10 patients (18.5%) had Musk antibody, 34 patients (62.9%) had acetylcholine receptor antibody, and 10 patients (18.5%) had none of these antibodies.

In this study, there was a significant relationship between Musk antibody and paresthesia (p value = 0.05). 

In the electromyographic findings, 38 patients (70.37%) had positive findings, 16 patients (29.6%) had normal findings.

In this study, 14 (25.9%) patients had ocular Myasthenia Gravis and the other 40 (74%) patients had generalized Myasthenia Gravis. In ocular Myasthenia Gravis patients, 2 patients (14.2%) had MUSK antibody, 5 patients (35.7%) had acetylcholine receptor antibody and 6 patients (42.8) had none of these antibodies. Among the generalized Myasthenia Gravis patients, 8 patients (20%) had MUSK antibody, 30 patients (75%) had acetylcholine receptor antibody and 2 patients (5%) had none of them.

**Table 2:** MUSK +/ - according to gender and type

	MUSK +	MUSK -	P VALUE
GENDER			0.68
female	7 (12.96%)	24 (44.4%)	
male	3 (5.5%)	20 (37%)	
TYPE			0.31
ocular	2 (14.2%)	12 (85%)	
generalized	8 (20%)	32 (80%)	

## Discussion and conclusion

This study aimed to investigate the characteristics of demographic, clinical, laboratory and electromyography in patients with Myasthenia Gravis, who referred to the neurology clinic of the Rasoul Akram hospital. In this study, 54 patients with a certain diagnosis of Myasthenia Gravis were studied. The average age of the patients was 47.3, with a standard deviation of 18.2, which in similar studies, was 37.6, with a standard deviation of 11.4, which generally, did not differ much as compared to the other studies [**7**].

The results of this study indicated a significant statistical difference between the average age of men and women, in fact, men had the disease in older age, and in older age, the symptoms emerged and the diagnosis would be established at a later age. The issue has been confirmed in other studies [**5**]. In this study, 57% were females and 43% were males, the male to female ratio was 1:1.3, that in other studies, 1:1.6 has been reported and all similar studies reported a higher proportion of women [**7**,**1**].

The most common symptoms of patients were ocular symptoms - 64.8%, followed by muscle weakness - 61%, while in other studies, the most common symptom after ptosis and diplopia was pharyngeal symptoms and the muscle weakness which was placed on the third place [**7**]. It should be noted that paresthesia and gait problems were the least important signs.

According to the laboratory data of the cases, 10 patients (18.5%) had antibody Musk, 34 patients (62.9%) had acetylcholine receptor antibody, and 10 patients (18.5%) had none of these two antibodies. According to the findings, this antibody has been reported positive in 35.7% of the patients with ocular myasthenia and also 75% of generalized myasthenia gravis patients [**5**]. MUSK antibody was more positive in females than in males and female/ male ratio was 2.3:1. It also seemed that generalized myasthenia MUSK antibody was more positive than the ocular Myasthenia Gravis but there was no meaningful statistical relation between the MUSK antibody and the sex of the patients or the muscular involvement type.

There was a history of thymectomy in 40% of the patients in this study, which had no significant difference in comparison with other studies; of course, the other studies represent the role of racial differences, for thymectomy with Asian race preference [**5**].

In the electromyographic findings, 38 patients (70.3%) had positive findings and 16 patients (29.6%) had normal findings. Among the generalized Myasthenia Gravis patients, 46% and, in ocular myasthenia, 30% had positive findings. According to Dr. Nafisi’s paper from 2012, normal EMG is more common in ocular patients especially when the patient’s lab data show negative results for antibodies and our results were the same [**6**,**10**].
